# Interpretations on the Diagnostic Dilemma in Thyroidology: Diffuse Large B‐Cell Lymphoma of the Thyroid With Hashimoto Thyroiditis

**DOI:** 10.1002/ccr3.72225

**Published:** 2026-03-06

**Authors:** Ilker Sengul, Demet Sengul

**Affiliations:** ^1^ Thyroidology Unit Giresun University Faculty of Medicine Giresun Turkey; ^2^ Division of Endocrine Surgery Giresun University Faculty of Medicine Giresun Turkey; ^3^ Department of General Surgery Giresun University Faculty of Medicine Giresun Turkey; ^4^ Department of Pathology Giresun University Faculty of Medicine Giresun Turkey

**Keywords:** diffuse large B‐cell lymphoma, fine‐needle aspiration cytology, immunocytochemistry, lymphoma, thyroid gland, thyroidologists, thyroidology

Dear Editor,

We are writing in response to the recent report in thyroidology, entitled “Diffuse large B‐Cell lymphoma of the thyroid in a patient with Hashimoto thyroiditis: a diagnostic dilemma,” presented by Karki et al. [1], published in *Clinical Case Reports*. This account demonstrates a stark illustration of the diagnostic intricacies inherent in primary thyroid lymphoma (PTL) when confounded by underlying Hashimoto thyroiditis (HT). Indeed, the authors have documented the limitations of fine‐needle aspiration cytology (FNAC) [2, 3, 4] in such ambiguous scenarios. The patient, an elderly man in his early seventies, presented with a progressively enlarging neck mass over suggested only lymphocytic thyroiditis, Category II. This reliance upon FNAC, despite persistent growth, is challenging, as systematic reviews report its sensitivity for PTL diagnosis as low as 48%. The necessity of subjecting the patient to a right hemithyroidectomy for definitive histopathological confirmation of diffuse large B‐cell lymphoma (DLBCL) underscores the diagnostic delay. Whilst the authors acknowledge that advanced diagnostic techniques are often inaccessible in low‐resource settings, it remains paramount that clinical suspicion guides the course of action. The case correctly advocates for the use of core needle biopsy (CNB) [5] and immunohistochemistry (IHC). CNB, by preserving tissue architecture and offering a higher yield, has superior diagnostic value, holding a sensitivity of 94.3% for PTL, and demonstrably reducing the requirement for diagnostic surgery. We must ensure that FNAC, though a standard first‐line tool, is not trusted implicitly when the clinical presentation—such as a rapidly enlarging mass—remains discordant. The diagnostic pathway in suspicious cases must include CNB to assure early and accurate subclassification, thus avoiding the undue morbidity attendant upon excisional biopsy when treatment necessitates systemic chemotherapy (R‐CHOP). The key clinical message may be that a high clinical suspicion of PTL, despite equivocal fine‐needle aspiration results, warrants prompt transition to core needle biopsy. This approach, offering 94.3% sensitivity, may ensure accurate subclassification and might enable immediate initiation of chemotherapy, thereby avoiding the morbidity associated with unnecessary surgical excision in these aggressive cases. We thank Karki et al. [1] for their work on DLBCL versus HT in *Clin Case Rep*.

## Author Contributions

**Ilker Sengul:** conceptualization, formal analysis, investigation, methodology, project administration, resources, software, validation, visualization, writing – original draft, writing – review and editing. **Demet Sengul:** conceptualization, formal analysis, investigation, methodology, project administration, resources, software, supervision, validation, visualization, writing – original draft, writing – review and editing.

## Funding

The authors have nothing to report.

## Ethics Statement

The authors have nothing to report.

## Conflicts of Interest

The authors declare no conflicts of interest.

## Data Availability

The datasets used and/or analyzed during this study are available from the corresponding author upon reasonable request.
